# OPTORER: A Dynamic Routing and Touring Service for Indoors and Outdoor Tours

**DOI:** 10.3390/s24082431

**Published:** 2024-04-10

**Authors:** Constantinos Vassilakis, Maria Polychronaki, Dimosthenis Margaritis, Dimitrios G. Kogias, Helen C. Leligou

**Affiliations:** 1 Department of Electrical and Electronics Engineering, Campus of Ancient Olive Grove, University of West Attica, 122 41 Egaleo, Greece; convassilakis@uniwa.gr (C.V.); m.polychronaki@uniwa.gr (M.P.); dimikog@uniwa.gr (D.G.K.); 2 Department of Industrial Design and Production Engineering, Campus of Ancient Olive Grove, University of West Attica, 122 41 Egaleo, Greece; auto45184@uniwa.gr

**Keywords:** routing service, UX optimization, thematic tours marketplace, content creation, public safety and wellbeing, dynamic routing, smartphones, wearables

## Abstract

This paper introduces a new routing and touring service both for outdoor and indoor places of touristic and cultural interest designed to be used in the wider area of Attica, Greece. This service is the result of the work performed in OPTORER (OPTORER: OPtimal rouTing and explOration of touRistic and cultural arEas of interest within Attica given personalized adaptive preferences, promoted underlying purpose, and interactive experience), project, and it aspires to offer a range of innovative and thematic routes to several specified points of interest in the selected area of Attica, encouraging the combination of indoor and outdoor routes in a single tour. The aim is to optimize the user experience while promoting specific, user-centric features, with safety and social welfare being a priority for every designed tour, resulting in enhancing the touristic experience in the area. Using a common smartphone device, as well as common wearable devices (i.e., smartwatches), the OPTORER service will provide an end-to-end solution by developing the algorithms and end-user applications, together with an orchestration platform responsible for managing, operating, and executing the service that produces and presents to the end user results derived from solving dynamically complex optimization problems.

## 1. Introduction

Tourism is one of the main pillars of the global economy. At the same time, social distancing has recently become one of the most pressing social problems due to the COVID-19 pandemic, since it introduced periods of mandatory confinement, which resulted in a social chain reaction in terms of travel, whether for entertainment or other reasons. And while tourism has been the most stressed sector affected significantly by the pandemic, it has managed to recover and see exponential growth in the past 1–2 years.

OPTORER is a research project [1] that aims to create a platform to support and enhance the touristic experience initially in the region of Attica, Greece. To achieve this, OPTORER introduces *a novel routing and exploration service in outdoor and indoor areas* of touristic and cultural interest in the broad area of Attica. OPTORER’s service falls under the category of Smart Tourism Technologies and Applications, covering a wide list of offered functions that are considered attractive to a tourist while introducing several new, innovative features for user satisfaction and psychological motivation.

To achieve this, there are three main technical challenges that the OPTORER service manages to address and provide efficient solutions to. These challenges are:

- *Indoor location and positioning*: GPS technology has made outdoor positioning very successful and widely supported. However, due to signal attenuation caused by building materials, indoor positioning systems cannot rely on this technology for efficient measurements. At the same time, many indoor positioning systems have been developed based on a wide range of technologies, including WLAN, infrared, ultrasonic, and Bluetooth [2]. Indoor location and positioning are made possible through the use of techniques such as trilateration, triangulation, and fingerprinting [3], which calculate distance using the strength level of a signal received by a user device. The signal is generated by multiple radio beacons placed indoors, while the relative position is decided based on the accumulated distance from the beacons. Bluetooth radio beacons covering indoor spaces can provide a low-cost, low-power solution, while a smartphone can act as a receiver. The accuracy achieved in positioning and tracking is critical for OPTORER service [4]. The research results for the use of BLE beacons for indoor routing will be discussed in this paper.
- *Physical user state assessment:* The physical effort exerted, or the physical state of a user at a particular moment, can be assessed using algorithms that have as input heart rate monitoring data and/or cadence/pace measurement, obtained from sensors embedded in smartphones and wearable devices. However, accuracy in estimation is really a challenge. In addition, estimating user experience from these data are a step forward in this type of evaluation as it attempts to capture real-time user sentiment regarding user satisfaction [5]. The results of applying a technique for physical user state assessment will be discussed in this paper.
- *Dynamic Multi-Criteria Routing Optimization*: The exact solution (i.e., finding a globally optimal solution) of the routing optimization problem will make it impossible to provide near-real-time results, as the routing problem belongs to the class of optimization problems known as NP-Complete, which means that it is not possible to quickly find an optimal solution, as the complexity increases significantly as the destinations involved in routing increase [6]. The need to develop *efficient meta-heuristic algorithms* to provide a near-optimal solution to the multi-criteria optimization problem in near-real time is a challenge that has been addressed in OPTORER application. Furthermore, due to the dynamic nature of a tour, the service is required to be able to partially solve the optimization problem when some of the variables change in order to provide updated results immediately [6]. The design of a metaheuristic algorithm that dynamically optimizes routing, along with the constraints that are agreed upon, is discussed in this paper.

Another additional challenge is the study of how to scale the service to a large number of users, especially considering the complex algorithms that should be executed in real-time per user for a tour. This includes not only the demand for high-performance algorithms but also for highly efficient management of infrastructure resources.

In general, OPTORER’s platform aims to combine the solutions to the three technical challenges: indoor and outdoor localization (using BLE beacons and GPS), physical assessment (using sensors found in common equipment like mobile phones and smart watches) and dynamic multi-criteria routing (designing and applying a meta-heuristic algorithm for solving the optimization problem) in one system. The system aims to enhance the experience of tourists visiting the area of Attica, Greece. An important role in this lies in the fact that the service can be used by professionals to add content (in the form of guided routes) that they could sell in the application’s marketplace. This way, a tourist will receive professional guidance by visiting the route at their own pace and time.

For the rest of this paper, the structure of the work is the following: in Section 2, discussion of related work in the literature takes place, while in Section 3, details about the design of the system are provided with emphasis on the main sub-systems that are needed. In Section 4, more details about the system architecture and the solutions to the technical challenges discussed above are provided. In Section 5, the results from the piloting events of the project, related to the system and measured module performance, are presented, while in Section 6, a discussion of future steps in expanding the work is presented. Finally, Section 7 presents conclusions.

## 2. Related Work

With COVID restrictions lifted, people have returned to their usual habits, and traveling is one of them, making tourism one of the sectors with the biggest growth after the pandemic. This growth has been supported by technological advancements, as more and more attention is given to applications and systems that aim to bring the tourism sector (e.g., hotels, tours, museums) closer to the digital era, offering an enhanced experience to citizens but also advantages to professionals. More specifically, in [7], the authors are focused on ‘Smart Tourism’, carrying out a thorough investigation of the technological developments in the field in the years 2013–2019. By examining more than 50 different bibliographic sources, they explain that during this time, specific technical terms have emerged, such as ‘Smart Tourism’ and ‘Smart Tourism Destination’, which express the citizens’ need for technological support, elevating their tourism experience to another level at each destination. Analyzing their findings in 12 technological pillars (e.g., Social Networks, Internet of Things and User Experience), they also list proposed Smart Tourism systems, of which a large percentage are navigation and destination recommendation applications (i.e., restaurants, museums, accommodation, etc.). An important role is played by the fact that these applications can be accessed by tourists through their mobile devices, adding further immediacy and personalization to their experiences.

Additional findings that prove the above are reported in [8], where the authors, in their attempt to classify the sectors in Smart Tourism research, refer to numerous reports of Smart Tourism Technologies (STTs). These focus on optimized tourism experiences, consumer satisfaction, and behavioral mobilization. In fact, among the systems preferred by tourists, they cite as typical examples of Smart Tourism adoption experience organization applications (e.g., determining routes, planning points of interest), but also handling procedures (e.g., completing payments, booking seats and tickets).

Finally, in [9], the authors are conducting research on the adoption of mobile technologies and applications in Smart Tourism, focusing on the demand for Smart Tourism applications from the consumer side. Their results indicate that there is increased satisfaction as well as psychological motivation for tourists when they are given the opportunity to act through their mobile device and have as many services as possible available at their ‘fingertips’. Especially attractive for tourists are applications related to accommodation and catering, applications with travel services (tickets, securing seats), and last but not least, applications for browsing and providing information about destinations.

To be able to provide a system that offers a high-quality service, OPTORER deals with certain technical challenges that have attracted the attention of the research and academic communities. More specifically, indoor positioning has played an important role for OPTORER, and it is a topic that has been researched, and the use of Bluetooth (BLE) technology has gained attention [10]. In general, an important design choice when designing a localization system, as in OPTORER, is the selection of the beacon type. Beacons can be categorized as Optical, Sonic, and Radio Frequency-based.

Optical systems are superior in terms of positioning, with a single-digit centimeter error being achievable. Additionally, the beacons can take the form of simple visible marks, eliminating concerns regarding power consumption. However, visual obstruction of said beacons is a major concern, as is the visual intrusiveness of optically based systems, given that any beacons/markers used, regardless of whether or not they emit in the visible spectrum, have to be placed in visible locations. In addition, when used with mobile phones, the camera of the device has to be constantly switched on and pointing towards markers.

Sonic systems are versatile and can be deployed in a variety of environments, including those where visual obstructions may be present. Relying on sound waves for localization, they are also less visually obstructive. Beaconless handheld transceiver-based systems exist and have been demonstrated to exhibit decimeter-level accuracy [11]. However, contemporary cell phones lack such sensors. Thus, the integration of such a system into a smartphone application would require that a peripheral be handed out to users and plugged into the user’s handset, rendering ultrasonic solutions unsuitable for tourism-oriented applications.

Radio frequency sensors in smartphones are not capable of receiving and processing arbitrary signals; they are integrated into transceivers for various existing protocols, such as IEEE 802.11 (Wi-Fi), Bluetooth, and Bluetooth Low Energy (BLE). Wi-Fi yields results comparable to our own in terms of error [12]. However, Wi-Fi is not intended for such applications, having orders of magnitude higher power consumption than BLE, which boasts beacon battery life in the order of a year. Thus, BLE was chosen as the only **non-intrusive**, **low maintenance option that relies on sensors present in a typical smartphone**. More details about the technologies that are available for indoor localization can also be found in Section 4.1.1.

Regarding the technical challenge of estimating the physical condition of a person based on common sensors found in smart watches or smartphones, the research focused on how to measure Activity Monitoring (AM). AM is a well-established technique for quantitatively assessing the intensity of Physical Activity (PA) undertaken by an individual over time. AM has been used in various cases of obesity [13], Chronic Obstructive Pulmonary Disease—COPD and mental health [14]. It is often used alone, or alongside traditional methods based on questionnaires or interviews, to reduce inaccuracies, such as recall bias, inherent in these qualitative techniques [15,16]. Devices used for AM often rely mainly on accelerometers, which measure the acceleration of the part of the body they are worn on. More details on this challenge can be found in Section 4.1.2.

## 3. System Design

Solutions to the technical challenges presented above are key in order for OPTORER to be able to meet its main objectives. These objectives are:

✓ To offer a routing and tour planner service in outdoor/indoor places of touristic and cultural interest, able to adjust to achieve specific purposes (personalized criteria and/or promoted purposes).
✓ To expand the number of indoor tours requiring only a low-cost initial investment from the operators of places of interest using BLE beacons.
✓ To assess the user experience and drive the dynamic adjustment of routing decisions along the tour.
✓ To provide the current state with the ability to ensure in real time the safety and well-being of citizens by communicating notifications or alerts to be taken into account in the dynamic routing decision process.

Taking into consideration the possible technical solutions that could be used in order to meet OPTORER’s objectives, the system is designed to include four main subsystems:

1. User Applications subsystem
2. Development subsystem
3. Controller subsystem
4. Infrastructure subsystem

Each of these subsystems plays a crucial role in the performance of the overall OPTORER system. In Section 3, details about the interconnection of the subsystems are provided to describe the overall system architecture. Here, a description of the functionality that each one brings to the system is given. 

### 3.1. Description of OPTORER’s Sub-Systems

The “*User Applications*” subsystem consists of two applications: a *mobile application* that enables the end user to navigate and customize an offered thematic tour using their smartphone or setup a new tour by selecting favorite points of interest through the app. The guidance of the user will be continuous, no matter if it is in an external or internal space, as long as the latter supports the OPTORER service with the required infrastructure (as explained later). To enable the switch between the two routing modes (they are not used simultaneously), whenever the user is tracked to enter a building that is equipped with the needed infrastructure, a prompt will appear on the user’s mobile phone to initiate the loading of the interior map. The mobile application also offers user experience assessment functionality, which will run continuously during the tour as long as the user wears a compatible wearable device (i.e., a smartwatch). This functionality collects data from the mobile phone sensors and the smartwatch in order to assess the physical state of the user. The heart rate information accurately provided by wearable devices is used, while additionally, the estimation of steps, which can be extracted both from the wearable device and from the mobile phone, is also monitored. The calculations are performed on the smartphone, delivering fast estimation and notification delivery, and these data are not stored in respect of the General Data Protection Regulation (GDPR) (The EU general data protection regulation (GDPR) governs how the personal data of individuals in the EU may be processed and transferred) rules in Europe. Any provided feedback/assessment from the user (e.g., the need to rest) will be delivered to the Controller subsystem for real-time, dynamic changes to the provided route with the aim of optimizing it. There is also a *web application* that is used to manage the users of the service and to add and manage the content that is offered. For example, for an indoor tour, the user can access the web application to draw a map of the interior place and to add important points of interest inside it. To allow such an action, the user should have a specific role. The role is also assigned and monitored by the web application. More details about the roles are provided in Sub-Section 3.2.

The “*Development Subsystem*” implements *the management of users and content* of the service while, per user and selected tour, it configures the initial conditions and constraints of the routing problem. The solution to the routing problem is performed dynamically and according to the user’s choices, the real-life reported changes in the conditions of the tour, and the monitored and assessed experience of the user. The routing problem is continuously resolved by the Controller Subsystem, and the results are fed to the mobile application to guide the user on his or her tour and navigate him/her through a selected number of Points of Interest (PoIs). In general, the Development subsystem includes five components: the “*User Management System*” component that implements the functions offered through the Web application involving the management of users and the management of the subsystems. These functions are allowed for the user in the role of *Administrator*. Additionally, there is the “*Content Management System*” component that implements these functions that are offered through the Web application concerning the user role-based management of the content. These functions are available for the user in the role of *Navigator* or *Cartographer* or *Service Partner* or *Feedback Provider*. In the “*Repository of points of interest and routes*” component the processed content concerning Areas of Interest (AoIs) or Points of Interest and routes is made available at different levels of detail, as required per case (e.g., a full map of the AoI if it concerns an equipped interior with a series of radio beacons). The Content Management System and the Repository of PoIs are the components used to create and configure a structured tour that is made available in the *Tours Marketplace* component. Finally, the “*Service Deployment Editor*” component is responsible for initializing the routing problem with those data and constraints, as configured by the selected tour and/or the user’s choices and preferences submitted by the User Application. It is the point of contact with the Mobile Application, both for guiding the user through a tour as a product of solving the routing problem in the Controller Subsystem and for dynamically configuring the data and constraints governing the tour as a result of continuously evaluating user experience from the User Experience Assessment functionality, but also from changes in the surrounding conditions because of unforeseen events while touring.

The “*Controller Subsystem*” is at the heart of the OPTORER System. It manages and monitors all the devices and resources of the service, as well as the changing conditions that dynamically shape the routing in a tour, altering the parameters in the multifactor optimization problem that is being solved in this subsystem for each user and each tour. To deliver this performance, the Controller subsystem consists of several components. Starting with the “*Service Deployment Manager*” which is responsible for receiving and forwarding the conditions, regarding each user’s tour, of the route problem to the “*Reasoning and Rules Engine*” component. The “*Optimization Engine*” is the component that runs the code of the applied model for the optimization of the tour problem, which uses selected meta-heuristic algorithms. The problem gets dynamically updated and enriched by the rules and constraints retrieved from the *Reasoning and Rules Engine* component. Additionally, the Reasoning and Rules Engine forms a collection of rules and restrictions that are stored in a structured way. Here, there is also the “*Internet of Things (IoT) Devices Manager*”, which is responsible for constantly collecting and providing information related to and retrieved from the available IoT devices (e.g., radio beacons, wearable devices, smartphones) in the system. It is also responsible for their management. The data that is collected includes information about the location of the user, such as the geographic location of a mobile device (outdoors) or its location relative to a radio beacon in the case of an indoor location, as well as other information such as the user’s current assessment of experience or, more fundamentally, its network connectivity. At the same time, there is the “*Virtual Infrastructure Orchestrator*”, who is responsible for continuously monitoring the system’s needs for resources and adapting the offered resources depending on demand. The last component is a publish—subscribe broker mechanism that exists to deliver notifications that are received from the IoT devices (e.g., alerts regarding the physical condition of the user that should trigger a dynamic recalculation of the touring route).

Finally, there is the “*Infrastructure Subsystem*”, which includes all the actual IoT devices (e.g., Beacons, Smartphones and Smartwatches) and the Cloud Resources that belong to the Infrastructure as a Service (IaaS) solution. 

### 3.2. Types of Users and Allowed Actions

To complete the design of the OPTORER system, the different user roles and the actions each one of them is allowed to perform are presented in this sub-section.

The basic user roles are:

- *End User:* The end user of the service uses the Mobile Application to follow a selected tour (pre-planned or custom). Users of the Mobile Application are able to search among pre-planned tours filtered by the category of interest, the cost, or the available time. Additionally, end users can create their own tour by setting the following details: the starting point, a specific order for visiting the already-registered PoIs, the endpoint, and the preferred means of transportation. In this case, PoIs can be selected using a filter based on the category of interest. Also, end users can use the mobile application to report an event at the point where they are, followed by a short description of the event.
- *Administrator:* The Administrator can define the technical parameters of the system, gain access to data recorded for security reasons (e.g., unsuccessful login attempts), and manage users (i.e., in terms of access role and status). There are also certain administrative functionalities that have specific roles assigned to them (i.e., administrator sub-roles). These roles are:
  ○ *Partner’s Content Management*: Core Functionality of this role is to review the content/services that partners intend to include in the system. Examination of the content offered (e.g., texts and photos) is essential on online platforms to avoid malicious content and also to avoid listing mistakes by partners.
  ○ *Event Management*: Core Functionality of this role is to review data related to events reported by end users or other sources (communication streams). Reviewing events before communicating them to end users is essential to avoid false alarms, which can cause unnecessary panic.
- *Tour Creator:* The Tour Creator creates and configures a tour, defining the sequence of visits to Areas of Interest as well as the sequence of visits to Points of Interest within the selected Areas. The tour can combine both indoor and outdoor locations. Additionally, the tour creator enters content related to the Areas and Points of Interest while setting tour scheduling and possible stops/visits to points related to external Partners Provided Services. This way, the Tour Creator can enhance the end user’s experience during the tour or cover possible needs along the way.
- *Cartographer*: The Cartographer creates maps of indoor spaces and defines the location of indoor PoIs. Also, the Cartographer utilizes the required radio beacon infrastructure as well as the topographical mapping of the area to define a tour inside the indoor area. Additionally, Cartographers can add more detailed descriptions to outdoor PoI that can be included in tours, combining the added indoor location.
- *External Service Provider*: The role of the External Service Provider can include offering services as well as adding points where these services can be found. These services can be included by each Tour Creator in any predesigned or custom tour. Given that the platform supports Business-to-Business (B2B) capabilities, a tour is enriched with offered services intended to be provided by third-party companies or freelancers. For example, at a museum, a point of interest can be added, pointing to a tour guide that offers guided tours. Accordingly, a travel agency could list its own recommended tour to be delivered through the OPTORER platform at a certain cost. A user can be added to this role by applying for registration by filling out a relative form.
- *Provider of feedback*: The Provider of Feedback role has the ability to provide feedback in relation to the service, the tours as a whole, or individual PoIs or professionals/services involved in the tour. The feedback can take the form of an evaluation but is also about providing relevant content. Every user of the service who participated in an offered tour will be able to provide feedback.

Finally, a user may have more than one role after being assigned the corresponding access rights.

## 4. System Architecture

In the previous section, a presentation of the four main subsystems that were designed to form the OPTORER System took place. Here, a discussion about the resulted architecture of the OPTORER System will be presented, followed by a presentation of the solutions to the main (research) challenges that were selected and the identification of the system component that performs the designed actions.

**Figure 1:**
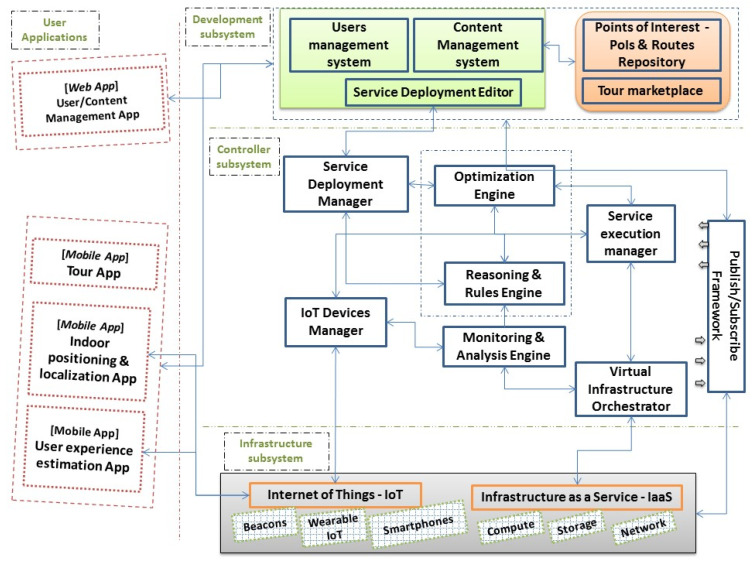
OPTORER Sub-Systems and their components.

**Figure 2:**
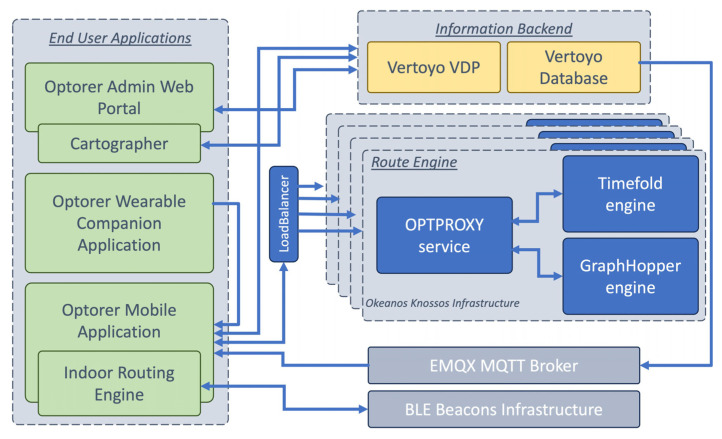
OPTORER final System Architecture.

Figure 1 depicts the final design of the OPTORER System Architecture, showing the components of each sub-system and the basic communications between them. Moving away from the design in Figure 1, the initially described sub-systems have received some minor adjustments and are highlighted by certain important tools, as seen in Figure 2. These tools and their interconnection with the rest of the OPTORER System are described here in detail for every subsystem:

- I “User Applications” subsystem consists of all the applications that a user of the system can access. There are three types of applications in general in OPTORER:
  ○ a web application that provides authorized users (i.e., those with the roles of Administrator, Tour Creator, Cartographer and External Service Provider) access to the Development subsystem. This access is provided through two different tools: the *OPTORER Admin Web Portal* tool and the *Cartographer* tool. The former allows for administration actions to take place along with any content management actions (e.g., adding, removing, or editing information for PoIs or AoIs) while the latter is the tool by which content managers can add the map of an indoor tour. This is conducted using the graphical environment available from the Cartographer tool in order to draw the floor plan of the interior space as well as to register the points where the BLE Beacons have been placed on the respective map. The last step of the process includes setting up Internal PoIs.
  ○ a mobile application, that is the main way in which the end user interacts with the OPTORER system and how (s)he can access and enjoy the functionalities and services offered by it. The mobile application has direct connectivity with all the other main tools in the other sub-systems (seen in Figure 2), including:
    - Connection to the wearable device application based on the Bluetooth protocol and using the native interfaces offered by its operating system.
    - Connection to the tools in the Development subsystem using the HTTP protocol and the available REST APIs, in order to obtain and configure the application’s relevant information for the user.
    - Connection to the Routing Engine tool inside the Controller subsystem using the HTTP RESTful interfaces offered by the corresponding GraphHopper tool [17].
    - Connection to the EMQX MQTT Broker tool in the Infrastructure subsystem, a real-time communication infrastructure using an MQTT client and subscribing to the channels that offer information related to emergency events that can affect the user experience. Here there is a change in the initial design (Figure 1), as the MQTT (pub-sub) broker is moved to the Infrastructure subsystem instead of the Controller subsystem since the emphasis is given to the communication of the information rather than the control and processing of it.
    - Connection to the Indoor Location Infrastructure tool using the Bluetooth protocol for continuous scanning of the available BLE Beacons and real-time positioning of the user on the indoor map.
    - Integration of Indoor Routing Engine functionality, using Dart language for smooth operation of the application. This tool is used to locate and position the user when (s)he moves in an indoor room. Also, the tool offers navigation using a pre-installed indoor map (by using the Cartographer tool).
  ○ A smartwatch application (OPTORER Wearable Companion Application) that provides information to the mobile application related to the user’s physical state. This is conducted locally by pairing the user’s watch with their mobile device using the Bluetooth protocol. This information contributes to the user experience exclusively and only in real time during a visit, while at any time, the user can, through the mobile application, delete these measurements from the memory of the application.
- The “Development subsystem”, or in other words the *Information Backend*, consists of two main tools (i.e., Vertoyo VDP and Vertoyo Database) that incorporate and deliver the actions and operations described in Section 3 for this subsystem. In more detail, Vertoyo Digital Platform (or Vertoyo VDP) is a platform based on the Java programming language that can be used to support complex web portals and mobile applications through a variety of ready-to-use features such as managing user roles/privileges and entity management/registration. It comes with advanced support functions, management of business workflows (Business Process Management-BPM), task scheduling, can be easily interfaced with Push/Email notification services, as well as with third-party systems, exposing or consuming programming interfaces such as REST or SOAP APIs. VDP communicates directly with the Vertoyo Database tool, a PostgreSQL DB that implements a custom SQL schema to store data for: (a) User Management-encrypted data for sensitive fields, (b) Content Management—static content of the Web application (e.g., PoI or AoIs), (c) Custom Entities Management—supporting data encryption. It is used to store the alerts coming from the MQTT pub-sub broker in OPTORER.
  The two tools inside the Development subsystem communicate with the web and mobile applications in the User Applications subsystem to give access to the back end to the certified users, and with the MQTT broker in the Infrastructure subsystem to receive updates that need to be stored in the system.
- The “Controller subsystem”, which is now called the *Route Engine* component, is a complex routing module that consists of three tools: the *OPTPROXY Proxy Application*, the *Graphhopper Routing Engine,* and the *Timefold Optimization Engine*. With the service implemented by the OPTPROXY proxy application, it is possible to communicate with the mobile application to submit the routing request and send a response to the user. Internally, the OPTROXY application takes care of communicating with the other two engine tools to return the optimal visit order if desired, as well as the optimal routing between them on the road network based on the user profile, the desired characteristics submitted by the user, and possible external constraints due to unforeseen events. In the final version, load scaling is achieved by installing the “Route Engine” as a set consisting of the proxy application, the Graphhopper routing engine, and the Timefold optimization engine on multiple virtual machines (Virtual machines) in a virtual server infrastructure and sharing the load on them (round robin).
- The “Infrastructure subsystem” consists of two tools: the EMQX MQTT Broker and the BLE Beacons Infrastructure. The former is a real-time information communication tool, implemented by an EMQX Broker. Through this, all the necessary information about any emergency events confirmed by the admin portal is transmitted to the end-user application on the mobile phone. In real time, this information is filtered based on the importance of the event, the current location, and the status of the user. For smooth and optimal operation of the platform, the real-time communication infrastructure is connected to two of the other tools of the system:
  ○ The mobile application for end-users allows for the dynamic and customized routing of the user based on emergency events.
  ○ The Information backend sends real-time and continuously updates the VDP database and the mobile application about the status of emergency events during browsing.

There is also the *BLE Beacons Infrastructure*, which is in fact independent of the rest of the system, while at the same time its communication is passive and is carried out exclusively by the end user’s Mobile Application. The infrastructure consists entirely of BLE radio beacons, which are installed indoors by the respective building managers. At the same time, with the help of the Cartographer tool during installation and in combination with the tools inside the Development subsystem, the user’s mobile phone can carry out all the functionalities related to the real-time positioning and navigation of the user in the corresponding interiors. It should be noted that apart from a difference in the visualization of the room, the experience is the same both for indoor and outdoor tours, meaning that the selection of points and the routing offer the same experience to the end user.

### 4.1. Technical Challenges

Having discussed the OPTORER System Design and (final) Architecture, it is time to delve deeper into the research and implementation decisions that were made to answer the technical challenges in OPTORER (see Section 1). The implementation of these solutions made it possible to meet the objectives of the project efficiently. 

In this sub-section, the solutions to the three challenges that OPTORER faced are presented in detail.

#### 4.1.1. Indoor Location and Positioning

OPTORER delivers a novel routing and exploration service that combines indoor and outdoor exploration in a single combined tour. While for outdoor routing, the use of GPS has been recognized as the go-to solution, things are not so straightforward regarding indoor technology, where the solutions are under research and receive continuous updates. 

Some of the more commonly used techniques for indoor locations include:

▪ Wi-Fi fingerprinting
▪ Inertial Navigation
▪ Bluetooth Low Energy (BLE) beacons
▪ Visual-based Navigation
▪ Magnetic Field-based Navigation

For the most part, the above methods are quite different from each other, as they use completely different equipment as well as a wide class of algorithms to place an object/user in an interior space.

In [18], the authors examine the use of metrics such as Wi-Fi Received Signal Strength Indication, Wi-Fi Round Trip Time, and visible signs, which are processed by trilateration, Fingerprinting with Neural Networks and Fingerprinting with Nearest Neighbor algorithm. Of the algorithms, the neural networks and Nearest Neighbor methods yielded maximum accuracy, with a small difference between them. Trilateration yielded an order of magnitude worse accuracy. A more detailed study on the trilateration method was produced in [19]. In this study, the authors describe the method of trilateration in detail. Next, they describe an experimental setup with three BLE transmitters in a room 9.0 m long and 10.2 m wide. Using a mobile device, they measured the error of the method at 163 points in the room. Their measurements were made with sample sizes of 1, 5, and 10 replicates and produced errors with means of 39.06 m, 8.96 m, and 7.97 m and medians of 8.04, 5.54 m, and 4.23 m, respectively.

On the other hand, Bluetooth Low Energy (BLE) is a communication protocol for Wireless Personal Area Network technology developed by the Bluetooth Special Interest Group (SIG). BLE, known as Bluetooth Smart, is part of the Bluetooth 4.0 standard: however, it has several additional functions compared to classic Bluetooth.

Indoor navigation using BLE radio beacons is an active area of research and development and is constantly evolving. BLE beacons are small battery-powered devices that transmit signals that can be received by mobile devices and have been used in a variety of applications, including indoor navigation.

Some of the current approaches to indoor navigation using BLE beacons include:

○ *Fingerprinting* [20]: This method involves creating a database of signal fingerprints at known locations within a building and using these fingerprints to estimate a user’s location based on signals received from nearby radio beacons. This approach has proven efficient in many indoor environments but can be computationally demanding, requiring frequent updates as the environment changes.
○ *Trilateration* [21,22]: This method involves using the signals from at least three radio beacons to triangulate a user’s location. The Free Space Path Loss (FSPL) formula is a popular method to be combined with trilateration since it converts the Received Signal Strength Indices (RSSI) to a given distance and then uses this as input to the trilateration process to convert distances from a known beacon location to coordinates. This approach is less computationally demanding than signal fingerprinting, but may be less accurate in environments with obstacles or other sources of interference.
○ *Machine learning* [23,24]: This approach involves training machine learning algorithms to recognize patterns in signal data and use these patterns to estimate a user’s location. This approach has the potential to be highly accurate and adaptable to changing environments, but it requires large amounts of training data and can be sensitive to changes in the signal environment.

In summary, any approach to the indoor location problem must strike a balance between accuracy and ease of implementation. At one end, there is the conventional solution of trilateration, which for two dimensions needs three transmitters and their coordinates. At the other extreme is the use of Fingerprinting, which requires a significant time investment to produce the measurements. In the case of data processing using neural networks, significant computing resources are also needed for their training. 

A middle ground is demanded for OPTORER, as some solutions do not provide enough accuracy to be useful, while others are extremely difficult to implement in the real world, as the required measurements are extremely time-consuming and any change in the layout of the space requires repeating them from zero. 

*The solution in OPTORER*: In order to identify the optimal technique for OPTORER, a solution that balances between accuracy and feasibility is followed, **the One Dimensional Fingerprinting (1FDP)**. Measurements that were conducted at the University of West Attica’s Ancient Olive Grove Campus, Hall ZA216, a rectangular room measuring 8.1 over 8.02m with three beacons located in well-known positions, have supported the efficient performance of the approach. 

Details about the methodology of the proposed solution and the evaluation results for the performance of 1FDP can be seen in [25]. 

**Figure 3:**
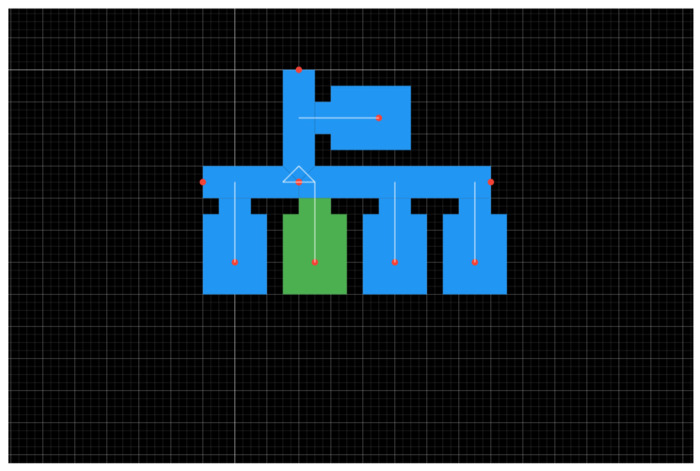
Recreation of an indoors space using the Cartographer tool.

Finally, it should be noted that for this service to be available to the end-user, a set of actions from the owner of the indoor space should take place in advance. First, the mapping of the indoor space should be designed using the Cartographer tool (Figure 3). There, the initial decisions to be made are the marking of spaces and the identification/assignment of the type of each space. The floor plan of each space is recreated through the repeating process of space marking by defining its shape and placing inside the space the corresponding emitters (Figure 3: green space is selected to add emitter information). After this step, the connections between the rooms are defined (e.g., mark any corridor) and the points of interest are also defined. The file is then uploaded to the Information Backend (see Figure 2) and is ready to be included in the design of a tour.

Another important step is the selection of the BLE beacons. The following two requirements exist for BLE beacons: the first is omnidirectional signal support, and the second is iBeacon protocol support. Additionally, the high advertising frequency is important. The next step includes the actual placement of the beacons inside the room. The placement of the beacons does not have to be in the exact location defined by the generated map, but it is recommended to use a trial-and-error approach to find an optimal point that produces a uniform signal with the smallest possible standard deviation. 

#### 4.1.2. Physical and Psychological User State Assessment

One of the goals of the OPTORER project is to assess the physical state of the user, which is one of the criteria that can influence the tour, even dynamically. For this purpose, OPTORER includes a companion application for smartwatches with the Android Wear operating system. Its creation was necessary to obtain the user’s biometric data, since the APIs of the Android Operating System for application communication with the user’s smartwatch require that the applications running on the two devices have the same ID. However, the purpose and functionality of the user applications and services cannot be entirely based on it, as it is not necessary that all end users own a smartwatch. Therefore, in order not to restrict the use of the mobile phone application only to users who wear a smartwatch, the functionality of physical and emotional state assessment is optional, but available to the respective users, who will have to install the application for wearables to activate the feature.

More specifically, the assessment is based on the *MET* (*Metabolic Equivalent of Task*) measurement, which is a new measure developed to provide information on how people consume energy during the day. MET is a measure used in dietetics and expresses the metabolic load of an activity in relation to the basic metabolic rate [26]. This measure takes into account energy expenditure during hours of activity and during hours of rest, offering a complete, detailed map of a person’s metabolic activity. Smartwatches and fitness trackers typically use their sensors (e.g., accelerometers, heart rate monitors) and other technologies, to record the user’s activity and biometric data. They then analyze this data to produce MET metrics such as the number of calories burned during the day, rest duration, and activity times.

The interpretation of these data are performed in the mobile application based on the calculation of the MET of the user’s walking and his pace [26]. It can be used to make qualitative inferences (if the user’s activity is considered vigorous exercise) or, in combination with user details such as gender, weight, and age, quantitative inferences (such as the user’s caloric intake). By knowing the user’s pace, energy consumption can be calculated, following the approach in [26]. By monitoring the result of this process, it is checked if the user has exceeded a threshold limit of 4 MET for the last 10 min, every 5 s. If so, the user receives a notification that gives them the option to take a shorter route if accepted. 

As a result, the use of this measure allows for the presentation of user data from the tour and to personalize his/her navigation based on the estimated physical condition without requiring the input of personal information. Finally, in order to comply with the General Data Protection Regulation (GDPR), the data is processed entirely on the user’s devices, and the results are not sent to the backend.

#### 4.1.3. Dynamic Multi-Criteria Routing Optimization

*Problem Statement*: The user starts an external tour from a geographic start point (START), with the final destination being a geographic arrival point (END), following a route that passes through a set of geographic points of interest (PoIs) and possibly a set of service points (STOPs). The START and END points can be the same (in case the user returns to his base after the tour) or different, while POIs or STOPs can be added by the user dynamically during the tour or even removed. The sequence of visiting PoIs (SEQ—Sequence) can be defined from the beginning (in the case that this serves a thematic tour) or free (NOSEQ—No Sequence). The user, depending on the Means of Transport (s)he uses or not (MoT—Means of Transport), is properly routed to his final destination (END) through the points (s)he wants to visit (PoIs, STOPs). The routes suggested to the user are selected based on criteria that indicate that they may be either the shortest in terms of distance, the shortest in terms of time, or those that enhance the user’s experience or allow better accessibility based on their abilities. Events (EVENTs) during a tour can dynamically reshape routing, making paths prohibitive in order to either protect the user, avoid their discomfort, or facilitate the community.

One of the PoIs that a user visits may be an indoor space, such as a museum, as long as the space is equipped with the BLE infrastructure described above. This allows the OPTORER mobile application to locate the user within it, with the user’s starting point being an internal PoI (ISTART—Internal Start) and destination internally to another, or the same point (IEND—Internal End), passing through a series of internal PoIs (IPOIs—Internal POIs) and possible internal stops where services are provided (ISTOPs—Internal STOPs). Corresponding to the outdoor tour, the indoor tour can follow a visiting sequence of IPOIs (ISEQ—Internal Sequence) or not (INOSEQ—Internal No Sequence), while an internal event (IEVENT—Internal Event), or an external event (EVENT) can dynamically configure internal routing. For example, an indoor fire will lead the user to the emergency exit, but so will an external event such as an earthquake.

The tour may be subject to time restrictions, either as a whole (TTIME—Total Time—time from the moment of starting to the final destination) or regarding the visit to an individual POI named X (VTIME(X)—Visit Time to POI X—time of visit to point X).

*Optimization Problem:* The optimization problem that was addressed in OPTORER, for each user navigating externally, is to *find the optimal route based on the criteria and constraints to be set*, starting from the starting point (START) and ending at the arrival point (END) by traversing a series of POIs and STOPs (service points), which can be dynamically configured by the user by adding new ones or removing already declared ones.

The available routes from one point (START, POI, STOP) to another point (POI, STOP, END) arise depending on the MoTs used or not by the user, as well as other restrictions that may have to be satisfied. The best possible of the routes, taking into account in the solution of the problem the set of individual routes, is selected based on the criteria set and is expressed by an *objective mathematical function*, the value of which must be optimal (minimum or maximum) for the selected total path among all possible paths. Indicatively, the objective function can express the total distance to be traveled, or the total time of the route, or the cost of the route, or some other metric that expresses the user’s experience during the route, or even a weighted combination of more criteria than those mentioned and/or others.

Constraints of the problem may include the visiting sequence (SEQ, ISEQ) of PoIs to be already predetermined. This can serve the purposes of a thematic tour, inside a predefined tour that the user chooses and that can be dynamically configured. Of course, the user can also deny the provided sequence for visiting PoIs. Visiting PoIs in a predetermined order reduces the complexity of the optimization problem since it significantly limits the number of available routes considered when selecting the optimal one.

*Modeling the optimization problem:* Multiple optimization problems have been extensively studied in the past in various versions, which are suitable and can be modeled accordingly to form related optimization problems like the one considered here. The benefit of such an approach is that it leverages years of research and development to fully understand the complexity of the problem as well as the behavior and requirements of various solutions, with the goal of deriving an optimal or near-optimal solution in a short time for “large” problems where the set of possible solutions to be considered is too many.

A well-studied routing problem that can be generalized and properly formulated to express the optimization problem under consideration is the **Vehicle Routing Problem (VRP)**. VRP is defined as the set of problems in which fleets of trucks are deployed in one or more depots. These fleets are required to serve a number of customers in pre-defined geographic locations. *The goal of VRP is to determine the appropriate routes to serve customers with the minimum cost* [27]. In classic VRP, each vehicle starts from a depot and returns to the same depot after uniquely visiting a set of customers assigned to it on its decided route. A wide range of variations of the problem have been studied in the literature. When considering one depot and one vehicle, *only then is the problem is the same as the well-known TSP (Travelling Salesman Problem)* [28], which was essentially the primary problem studied and from which VRP arose.

VRP belongs to the class of problems where the decision of the optimal solution is NP-hard [29], since there is no algorithm of polynomial complexity to solve the problem. This translates that VRP, as well as the problem addressed in OPTORER, belongs to a class of problems where the size of the problems (equivalent input size, e.g., number of customers, trucks, depots in the VRP) that can be solved by mathematical methods, or combinatorial optimization, is limited. This is because of the computing power, and thus the time required to find an optimal solution as the problem size grows becomes prohibitive (i.e., it exponentially increases with the problem size). Therefore, finding a globally optimal solution is time-realistic only for small-scale problems. However, a series of heuristics and metaheuristics algorithms that have been developed aim to find a good enough solution close to the optimal solution (i.e., a near optimal solution), if not the optimal solution, with an intelligent and non-exhaustive search for solutions in short times and for large problem sizes.

A *heuristic algorithm* is essentially a technique designed to solve a problem faster, while a *meta-heuristic* is a higher-level process or heuristic designed to find, generate, tune, or select a heuristic that can provide a reasonably good solution to an optimization problem. Popular heuristic/meta-heuristic algorithms are Tabu Search, Simulated Annealing and Genetic [29]. Variations of the VRP problem arise by considering different constraints taken into account and involving information on the characteristics of customers, vehicles, routes, delivered products, and drivers, as well as by considering different optimization objectives expressed by the appropriate objective functions.

The *mathematical formulation* of the classical Capacitated Vehicle Routing Problem (CVRP) concerns a fleet of K identical vehicles (of the same capacity C) making deliveries to customers from a central depot. CVRP can be described as the following graph theoretic problem: Let *G* = (*V*,*A*) be a complete graph, where *V* = {0, 1, …, *n*} is the set of vertices and A is the set of edges. Nodes *i* = 1, …, *n* correspond to the customers, while node 0 corresponds to the depot. A non-negative cost, *c_ij_*, is associated with each arc (*i*,*j*) ∈ *A* and represents the travel cost spent to go from node i to node *j*. We consider a directed graph, therefore, *c_ij_* ≠ *c_ji_.*

Each customer *i* (*i* = 1, …, *n*) is associated with a known non-negative demand, *d_i_*, to be delivered, and the depot has a fictitious demand, *d*_0_ = 0. Given a set of nodes *S* ⊆ *V*, let $d\left(S\right)=\sum_{i\in S}d_{i}$ denote the total demand of the subset of customers. From the vehicle routing problem, K vehicle routes should be generated, where each route will start and end at the depot and each vehicle will visit a subset of customers. Each vehicle will perform only one route, while all vehicles have the same capacity. Each customer must be visited by only one vehicle, while the total demands of customers visiting a vehicle on a route must not exceed its capacity. The specific routes will be chosen so as to minimize distribution costs [30].

Therefore, from the above results, the mathematical formulation of the problem, as presented as an extension of the TSP problem formulation by Dantzig, Fulkerson, and Johnson to create a two-index vehicle flow formulation in VRP:

$$min\sum_{i\in V}\sum_{j\in V}c_{ij}x_{ij}$$

subject to

(1) $$\sum_{i\in V}x_{ij}=1\;\;\;\forall j\in V\;\backslash \left\{0\right\}\;$$

(2) $$\sum_{j\in V}x_{ij}=1\;\;\;\forall i\in V\;\backslash \left\{0\right\}\;$$

(3) $$\sum_{i\in V\backslash \left\{0\right\}\;}x_{i0}=K$$

(4) $$\sum_{j\in V\backslash \left\{0\right\}\;}x_{0j}=K$$

(5) $$\sum_{i\notin S}\sum_{j\in S}x_{ij}\geq r\left(S\right),\;\;\;\forall S\subseteq V\backslash \left\{0\right\},S\neq \emptyset$$

(6) $$x_{ij}\in \left\{0,1\right\}\;\;\;\forall i,j\in V$$

In this formulation, *c_ij_* represents the cost of going from node *i* to node *j*, *x_ij_* is a binary variable that has a value of 1 if the arc going from *i* to *j* is considered part of the solution and a value of 0 otherwise, *K* is the number of available vehicles, and *r*(*S*) corresponds to the minimum number of vehicles required to serve (in terms of its demands) the set *S*. We also assume that 0 is the depot node.

Constraints (1) and (2) state that exactly one arc enters and exactly one exits each vertex associated with a customer, respectively. Constraints (3) and (4) say that the number of vehicles leaving the depot is the same as the number entering. Constraints (5) are Capacity-Cut Constraints (CCCs), which enforce that the routes must be connected and that the demand on each route must not exceed the vehicle’s capacity. The capacity cut constraints remain correct if *r*(*S*) is replaced by the lower bound $\left\lceil d\left(S\right)/C\right\rceil$ where *C* is the capacity of each vehicle and *d*(*S*) is the total customer demand. Finally, constraints (6) are integrity constraints. In the formulation, it has been assumed that the demands of any customer do not exceed the capacity of the vehicle.

The mathematical formulation of the problem has extensively been used to model the basic VRP (CVRP) and VRPB (VRP with Backhauls is an extension that includes both a set of customers to whom products are to be delivered and a set of vendors whose goods need to be transported back to the distribution center). However, it is considered suitable for these simple problems. It can be used only when the cost of the solution can be expressed as the sum of the costs of the arcs. We also cannot know which vehicle crosses each arc. Therefore, we cannot use it for more complex models, where the cost or even the feasibility of the solution depends on the customer order or the vehicles used.

We appropriately formulate the mathematical formulation of the classical VRP problem as we presented it in order to express the case where we have a single vehicle that has the ability to meet the demands of all *n* customers while it starts its route from a warehouse 0 and necessarily ends at a second warehouse *n* + 1, optimally visiting all n customers/locations. Essentially, in this version of the problem we are describing, the problem is that of the TSP traveling salesman, with the variation that he does not return to the point of departure but terminates the route at some other given point.

**Figure 4:**
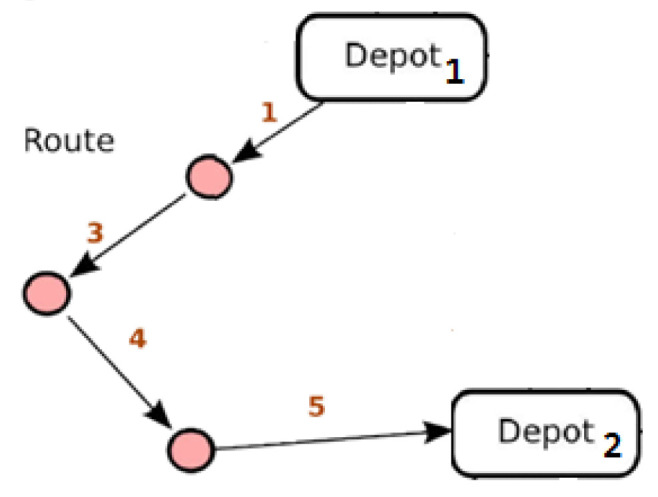
Illustration of the solution of the variant of the classical VRP problem with one vehicle and different arrival warehouses.

Figure 4 graphically illustrates the solution to the VRP variant we described without specifying the values of the problem parameters.

We set the number of available vehicles *K* = 1, the minimum number of vehicles to serve customers’ demands $r\left(S\right)=1$, the 2nd depot where the vehicle must end its route as depot *n* + 1 and modify condition (2) to express that the vehicle will terminate at depot *n* + 1 and not at depot 0 from which it departed. 

This results in the following mathematical formulation expressing the variant of the classic VRP we described:

$$min\sum_{i\in V}\sum_{j\in V}c_{ij}x_{ij}$$

subject to

(7) $$\sum_{i\in V}x_{ij}=1\;\;\;\forall j\in V\;\backslash \left\{0,n+1\right\}\;$$

(8) $$\sum_{j\in V}x_{ij}=1\;\;\;\forall i\in V\;\backslash \left\{0,n+1\right\}\;$$

(9) $$\sum_{i\in V\backslash \left\{0,n+1\right\}\;}x_{in}=1$$

(10) $$\sum_{j\in V\backslash \left\{0,n+1\right\}\;}x_{0j}=1$$

(11) $$\sum_{i\notin S}\sum_{j\in S}x_{ij}\geq 1,\;\;\;\forall S\subseteq V\backslash \left\{0,n+1\right\},S\neq \emptyset$$

(12) $$x_{ij}\in \left\{0,1\right\}\;\;\;\forall i,j\in V$$

**Figure 5:**
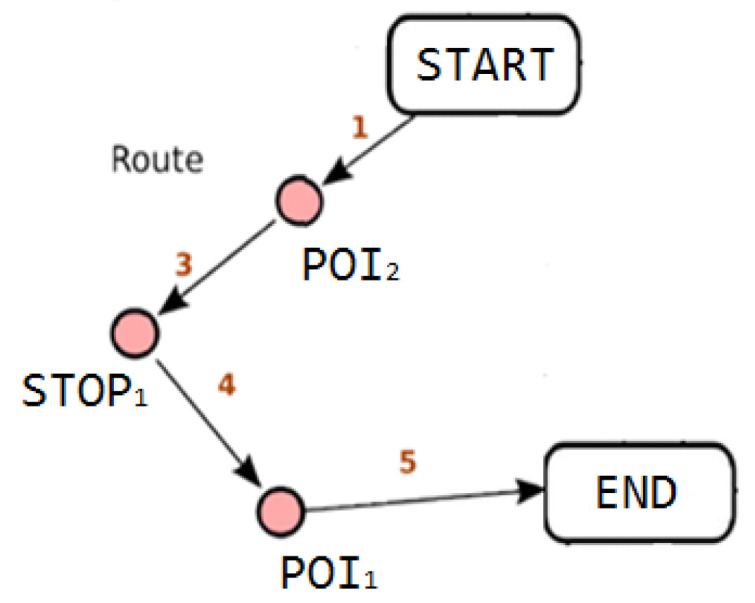
Illustration of the solution of the variant of the classic VRP problem with one vehicle and a different arrival depot with a direct mapping to the optimization problem we are asked to solve in the OPTORER service.

This variation in the classic VRP problem finds an exact correspondence with the optimization problem we are asked to address in OPTORER if we consider the departure depot as the START point (or ISTART internally), the route ending depot as the END point ((or IEND indoors) and customer locations as POIs or STOPs ((or IPOIs or ISTOPS internally). Figure 5 graphically illustrates the solution to an OPTORER service routing problem.

In the case that the sequence of visits is initially predetermined in whole or in part (e.g., the visit to POIx must be preceded by a visit to POIy), then the solution in whole or in part is known to the static problem. Thus, part of the solution as expressed by the parameters $x_{ij}\in \left\{0,1\right\}\;\forall i,j\in V$ is known and is introduced as a constraint in the problem formulation by setting the parameter $x_{ij}=1$ if the visit to node i must necessarily be followed by visiting node *j*.

The input to the problem should be the costs of the individual paths between all the nodes of the graph. Deriving the cost of a route from one node to another node depends on what it represents as well as whether or not the means of transportation are used. Illustratively, the cost may express the distance (depending on the MoT) between the nodes and/or the travel time (depending on traffic or other factors) and/or the user experience (based on an assessment of inconvenience or pleasure in a nice ride).

The derivation of the cost and the selection of the individual path from one point to another are also subproblems that must be solved before the optimization problem we are considering. Algorithms commonly used by services such as Google Maps to choose a suitable route on the map and based on other factors such as traffic are Dijkstra’s [31] and A* [30] for finding the minimum path in a graph where the edges have weights. 

The navigation of the user from one point to another on the path that has resulted from solving the optimization problem is a different function of the OPTORER system; however, the navigation should be conducted on the individual path that has been chosen based on cost.

In OPTORER service, the problem is not considered static but *dynamic*, where visiting points can be changed along the route according to the traveler’s desire, the MoT (e.g., a part of the route can be performed by car and then the browser continues on foot), some event triggers a change to the route or changes the costs of specific routes, or the tour from an outdoor area is continued in an indoor area that supports the technology introduced by OPTORER. The obvious thing is that any change triggers the system to re-solve the problem with the entry of the new data. However, given the complexity of the problem, techniques to partially solve it are considered with the goal of near-real-time decision making.

Considering the OPTORER service optimization problem as a variant of the VRP problem, rather than a variant of the TSP problem in the first place is a tactical choice, as future extensions of the service will be able to rely on an extension of the modeling and implementation to be conducted [32]. Possible extensions include adopting time windows, supporting mass transit for travelers, controlling the attendance of travelers using the service at points of interest such as museums based on capacity, etc.

*Optimization Engine Implementation:* OptaPlanner [33] is the choice for implementing the optimization engine of the OPTORER service. Apart from being a free software solution, OptaPlanner also provides excellent possibilities for solving optimization problems with heuristic algorithms (such as tabu search, simulated annealing), while enabling the solution to be easily integrated with a number of other modern technologies required to implement an optimization engine in the context of a high-scale service (e.g., openshift, quarkus, spring boot), while accepting input and providing output via APIs REST APIs in JSON format, which further facilitates its integration as a module of a system like OPTORER. In addition, it supports a number of techniques that facilitate solving dynamic problems in near real time (e.g., real-time planning).

The process of solving a problem with OptaPlanner consists of modeling the problem as a set of classes, appropriately annotated based on their role (PlanningSolution, PlanningEntity, etc.), defining constraints (as mandatory—Hard and flexible—Soft), the definition of the function to be optimized (the score function, as it is called), the selection and configuration of the algorithm that will solve the problem, the loading of the input data to the problem, and finally the execution of its solution.

OptaPlanner integrates well, as demonstrated in various demo applications, with open-source routing engines such as GraphHopper [17] (but also Google Maps), which is a map-optimized route engine using OpenStreetMap mapping [34], which provides open mapping data. GraphHopper 8.0 uses efficient algorithms to decide the route on a road network, offering three different options for algorithms for routing [35]: (1) the flexible mode, which uses well-known shortest path calculation algorithms such as Dijkstra or A*, which are still considered too slow for most real-world graphs; (2) the hybrid mode, which uses Landmarks (LM). The A* algorithm is employed with a heuristic based on landmarks and the triangle inequality for the goal direction. The algorithm is based on the election of a few landmarks, which can be any node of the graph, and distances calculated from these landmarks to all nodes of the graph as a pre-processing step; and (3) the speed mode, which implements Contraction Hierarchies (CH). Shortcuts (a shortcut connects two vertices not adjacent in the original graph) are created in a preprocessing phase and are then used during a shortest-path query to skip over less important vertices, achieving a significant speed-up in shortest path calculation. CH are provided with the graph as input and are able to assign importance to vertices using heuristics and create shortcuts. CH still employs a bidirectional Dijkstra or A* algorithm but works on a “shortcut graph”. Graphhopper can be configured and modified appropriately so that the individual route decision is according to the intended goals.

Also, excellent integration has been demonstrated for the visualization of results using Leaflet.js [36] and also Google Maps. For our solution, we adopt Timefold [37], which is the latest different path to the implementation of Optaplanner with exceptional refined characteristics, while we integrate it with Graphhopper. our choice among the offered routing algorithms is that of the CH algorithm, as it provides a significant speed-up in the calculation of the route while maintaining the desired quality characteristics regarding the path selection. 

**Figure 6:**
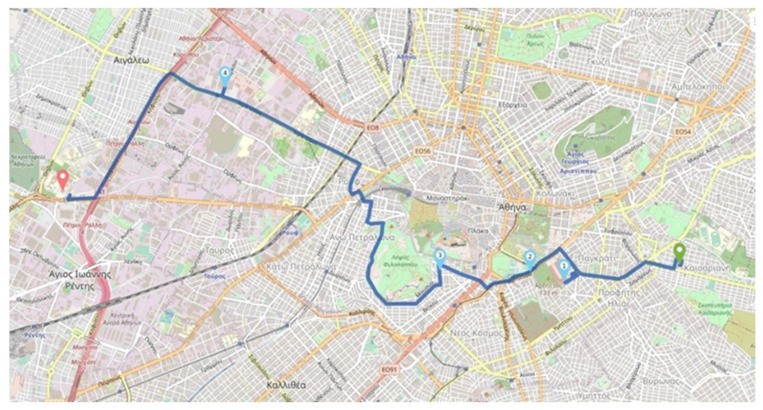
Indicative tour and road route in the city center of Athens, Greece.

Figure 6 illustrates an indicative tour on the map as a visual representation of the solution to an optimization problem we consider in OPTORER; the start point is the first on the right on the map, the arrival point is the last on the left on the map, and the intermediate points are visited in a sequential manner as defined by the numbers that signify them).

## 5. Evaluation Results

For evaluation purposes, tests for the main components of the OPTORER service were conducted. Therefore, evaluation took place for the Mobile Application and the Routing Optimization process. It should be noted that evaluation tests for the indoor localization and positioning service were conducted and can be found in [25]. Additionally, the reader could visit the OPTORER project YouTube channel and take a look and feel at the applications that have been developed (https://www.youtube.com/@OptorerPPEproject, accessed on 4 February 2024).

The results of the evaluation process are:

- *Mobile Application Evaluation Results*: During the test, there were certain KPIs that needed to be met in order to make sure that the performance of the modules was successful. Table 1 presents the KPIs that were tested during the evaluation of the mobile application, along with the results from the measurements that were captured:

For the Mobile Application, the related indicators were measured using either separate blocks of code running simultaneously with the application code or using one of three separate tools. These include the Firebase Performance Monitoring tool [42], offered by Google the New Relic Performance Monitoring Tool [43]; and the Android Studio [44] integrated performance measuring tool, which is called Flutter DevTools [45]. Lastly, due to the fact that battery monitoring requires very specific and native permissions given by the device, the battery consumption of the application was measured using the AccuBattery Application [46] during in-lab testing. A demo route (approximately 10 min in duration) was designed and carried through, including rerouting due to path drifting, during which the AccuBattery Application was activated on the same device.

The monitoring process of these tools has produced a number of charts showing the above-achieved values regarding mobile application memory and network performance. It should be noticed that for the KPIs referring to the start time of the application as well as the average battery consumption, the device’s hardware capabilities could significantly affect the performance, presenting high fluctuations. The performance monitoring charts are shown in Appendix A.

- *“Route Engine” Evaluation Results*: The solution to the routing problem is activated by a call providing the appropriate input data to the complex module that we call “Route Engine”. The Calculation of an optimal route is initiated, and upon completion of the solution, the output data describing the route to be followed by the user is returned. The route is initially calculated before the start of the tour and re-calculated whenever the tour or the conditions in which it takes place change.

The “Route Engine” consists of the OPTPROXY proxy service, the GraphHopper routing engine, and the Timefold Optimization Engine. The individual engines internally complete additional modules such as the OpenStreetmap implementation and the inference and rules engine. A call to find an optimal path for a tour is made to the OPTPROXY proxy service, where the request specifies whether a sequence of points defining the tour also matches the visit sequence during the tour or not. In the first case, the proxy application will send a query to the GraphHopper engine and return the response it will receive through OPTPROXY, while in the second case, it will first send an appropriately formatted query to the Timefold engine (version 1.5), and after receiving the response with the optimal visit order, it will appropriately configure the original request and forward it to the GraphHopper engine, from where it will receive the response that will be returned via OPTPROXY. The Timefold engine is only queried if the visit points are not given in their desired visiting order, and thus the order that minimizes the total distance traveled or total time spent to complete the tour must be found. It is clear that significant time will be spent in the optimization engine solving the computationally complex problem of finding the optimal visiting order whenever this is required.

In the following performance study, we conduct a worst-case analysis assuming that all requests refer to tours with a free order of visit, while each request is for the maximum number of visit points that we consider a tour may include. In the performance study, we consider and study, the instantaneous number of concurrent requests to find optimal routing. Such a worst-case study approach will lead us to a safe dimensioning, parameterization, and forecasting of the resources we must have available to continuously achieve our goals. In addition, the quality of the solution will be evaluated in the form of deviation from the optimal one in relation to the resources and time available for finding it.

In the following scenario, the correlations between load expressed in the number of simultaneous requests, computing resources, quality of the generated solution, and request processing time are studied. The results of the study lead to the optimal parameterization of the system and the individual routing and optimization engines so that within the targeted times and the targeted maximum number of requests, the best possible result is produced utilizing the available computing resources.

**Figure 7:**
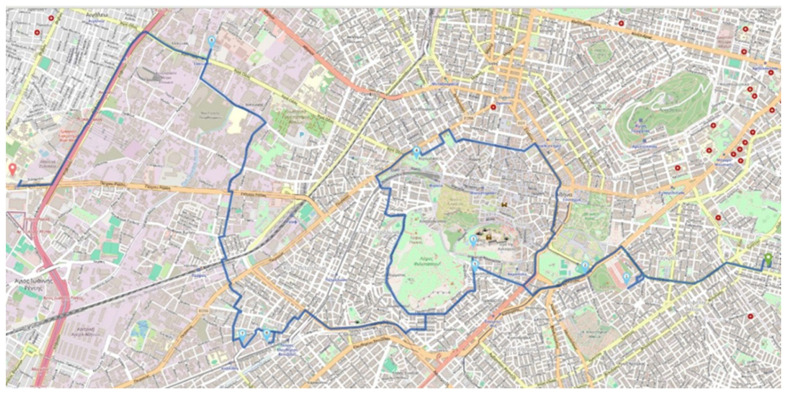
Trial tour used in our evaluation and visualization of the solution to the routing problem as a road route located in Athens, Greece.

The OPTPROXY application, the GraphHopper engine (with Attica’s roadmap), and the Timefold engine run on the same virtual machine provided by a rather stable infrastructure, exhibiting very low variation in the share of physical resources over time. We use a virtual machine with a processor (CPU) with eight cores (cores) and 16 GB of memory. In order to have a view of the available resources, we run the PassMark [47] benchmark. By studying the variability of resources over time by running the benchmark at different times, we conclude that the variability is low enough, which confirms the stability of resources over time. The PassMark CPU mark metric reflecting the computational power of the virtual machine has a value close to 8000, which ranks the virtual machine’s CPU computational performance in the mid- to high-performance CPUs [48]. The GraphHopper engine runs using the Attica road map. The Timefold machine is configured to give up to 5 s to solve each optimization problem, while stopping the solution if in 1 s it has not found a better solution than the previous solution it had found to the problem. The number of problems allowed to be solved by the engine at the same time (number of simultaneous solvers–solvers) is studied by setting as a value 8 (1 thread per core), 16 (2 threads per core), 32 (4 threads per core), 64 (8 threads per core), and 96 (12 threads per core). We use the same request describing a tour each time, which consists of a total of 10 points, including the starting and destination points, without a predetermined order of visit. We note that the problem is solved anew each time for each new request, while the selection of points for the tour has been made so that finding the route is classified as complex. Given the nature of our service, each tour requested by a user is expected to include from 2 to a maximum of 10 visit points, including the starting point and the destination. The trial tour is shown on the map in Figure 7 (start point first on the right on the map, arrival point last on the left on the map, intermediate visit points in a sequential manner as defined by the numbers that mark them). The route shown is a visualization of the solution to the routing problem.

We run each experiment once and present the results, considering that the variation we will have in the value of the considered metrics will be small in repeated experiments due to the exclusive use of the virtual server as well as the observed constancy of infrastructure resource performance. This claim was confirmed by multiple runs of the same experiment. The following table (Table 2) lists the minimum, average, and maximum request processing time achieved for different values of concurrent requests and active concurrent solver threads. The quality of the solution we achieve in all the cases we consider is the optimal one.

We observe that having 32 to 96 concurrent solvers provides for an average request processing time below 5 s, even in the case of 200 concurrent requests. It is clear that as the number of concurrently active solvers increases, the computational power available for a single request decreases. However, when concurrent requests exceed the number of concurrently active solvers, the requests not being able to receive service on arrival will be queued, waiting for service when resources will be available. So, there is a trade-off between the computational power a single request is able to receive and the queueing delay it may suffer in order to receive that computational power. One could think that the best decision would be to have as many active solvers as the number of CPU cores available in order for each optimization problem to obtain a good or optimal solution in less time. This is true when we consider quite larger instances of the problem (e.g., a tour with 200 points considered), requiring significantly more time for the given computational resources to be solved with a good solution provided. It is then that the queueing delay becomes less significant since the real tradeoff is between computational power, time, and the quality of the solution. In our case, given the computational resources available, the target times to provide a solution, the expected number of points a tour includes, and the maximum number of concurrent requests considered, we achieve an optimal solution to the problem at all times, and our concern is to find the number of active solvers and maximum number of concurrent requests a “route engine” is permitted to serve in order to provide the quality of service targeted. The scaling of the “Route Engine” complex module in the current version of OPTORER is achieved by sharing the load among several virtual servers hosting a “Route Engine” in a round-robin or weighted according to their computational power.

## 6. Discussion

While this paper focuses on the main technical challenges of OPTORER system, there are also some additional features that OPTORER service possesses that were not discussed in detail, but they add value to the overall user experience, like:

- the tours marketplace, allowing end-users to select preselected tours that were designed and offered for everyone to buy.
- the possibility for third-party service providers to add their service inside the system so that it can be selected either from a tour organizer in a pre-selected tour or from the system when it dynamically responds to a change in the conditions (either a physical condition due to fatigue or environmental conditions due to an accident).
- the reporting of incidents from the users and the assessment of the tours can help the system prioritize the available tours and act when necessary to provide alternate routes to the users that are affected by any dynamic changes to their tour.

Finally, for the future, possible extensions to the service may include the use of artificial intelligence (AI) to improve the physical assessment and the interconnection of the service with other services to receive alerts about the weather or emergency notifications that could demand an alert be provided to the affected users. Interconnection with public systems will also be an important step for this service.

Additionally, enhancements should also be in place in the future for the interior localization technique, which should be tested for a very large number of simultaneous users. Also, tests using more expensive beacons can also take place, even though there is a need to balance cost and accuracy (as discussed in 3.1).

Finally, for the physical condition assessment, the use of other sensors will also be investigated, as long as they are easy to find and to wear by the end-users. Further combinations of the selected measurements, to predict the current condition will be investigated using prompts from the user regarding his/her physical data (e.g., height, weight). These data can be used to provide more accurate predictions, possibly using artificial intelligence for this, as suggested earlier in this topic.

## 7. Conclusions

This paper presents the results of the design and implementation of a novel routing and touring service initially scheduled for the Attica region of Greece. The work was conducted for the OPTORER project, and in order to be delivered, certain technological challenges needed to be addressed. Indoor Routing, Physical Assessment, and Dynamic Multicriteria Outdoors routing were the three main challenges, and a description of the implemented solutions was presented here. The results of the main module evaluation were also presented and discussed.

## Figures and Tables

**Table 1:** Tested KPIs and Results for Module Performance.

KPI	Target Value	Achieved Value	Tool Used
User Location Time	<3 s (on avg)	<65 ms	Flutter Dart Code
User Location Accuracy	<1.5 m	~1 m	Flutter Dart Code
Time to load the Application	<3 s	<1.58 s	Firebase APM
Memory Usage in mobile phone during app execution	<=512 MB	<150 MB	Flutter DevTools
Data/min needed to follow a route in the mobile phone (on avg)	<=1 MB/min [38]	<500 KB/min	New Relic APM360
Time to place the user location on the map	<=20 s [39,40]	<4 s	New Relic APM360
Average battery consumption on the mobile phone	<=150 mAh [41]	<90 mAh	AccuBattery App
Time to load the map (Example: when moving from outdoors to indoors)	<=10 s	<3 s	Flutter Dart Code

**Table 2:** Results for the minimum, average, and maximum request processing time achieved for different values of concurrent requests (first bold column) and active concurrent solver threads (second bold column).

Concurrent Requests	Active Solvers	Minimum Request Processing Time (In Seconds)	Average Request Processing Time (In Seconds)	Maximum Request Processing Time (In Seconds)
**8**	**8**	1.25	1.28	1.3
**16**	**8**	1.28	1.76	2.23
**32**	**8**	1.07	2.44	3.73
**64**	**8**	1.32	4.51	7.79
**128**	**8**	1.32	6.86	14.69
**200**	**8**	1.32	9.53	21.42
**8**	**16**	1.27	1.29	1.32
**16**	**16**	1.27	1.33	1.4
**32**	**16**	1.16	1.76	2.36
**64**	**16**	1.24	2.42	3.57
**128**	**16**	2.21	4.85	8.51
**200**	**16**	2.56	6.54	11.16
**8**	**32**	1.27	1.28	1.29
**16**	**32**	1.27	1.31	1.37
**32**	**32**	1.29	1.50	1.93
**64**	**32**	1.12	1.82	2.48
**128**	**32**	2.31	3.27	4.87
**200**	**32**	2.36	4.55	7.44
**8**	**64**	1.28	1.29	1.3
**16**	**64**	1.28	1.30	1.33
**32**	**64**	1.18	1.52	2.25
**64**	**64**	1.1	1.81	2.5
**128**	**64**	1.38	2.67	3.63
**200**	**64**	1.72	3.55	5.76
**8**	**96**	1.25	1.27	1.29
**16**	**96**	1.24	1.26	1.3
**32**	**96**	1.25	1.39	1.55
**64**	**96**	1.19	1.91	2.74
**128**	**96**	1.77	2.54	3.28
**200**	**96**	1.37	3.60	6.53

**Figure A1:**
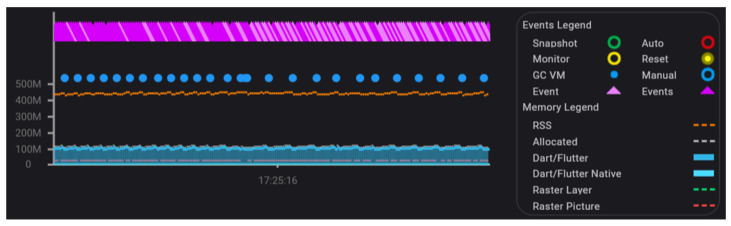
Android Studio DevTools: Optorer Application Memory Performance (Bottom Blue Graph—Dart/Flutter).

**Figure A2:**
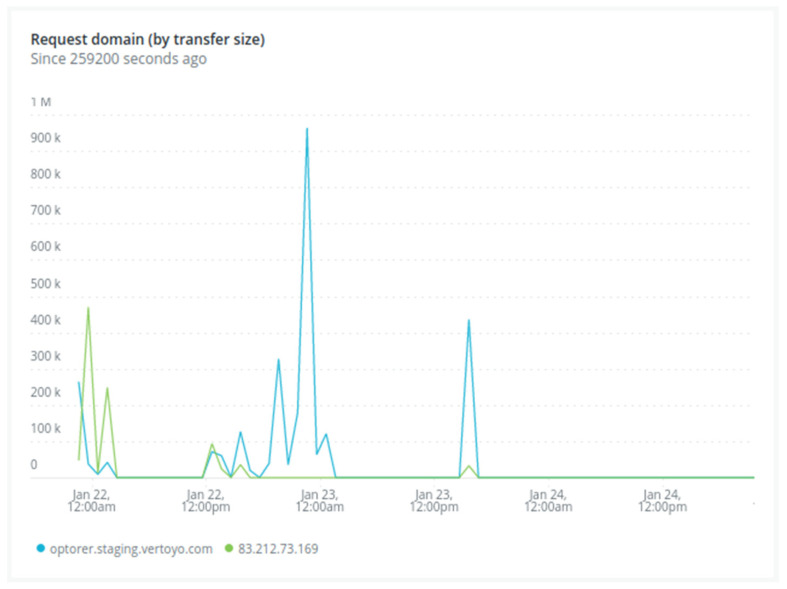
Average HTTP Transfer Size during navigation (green graph).

**Figure A3:**
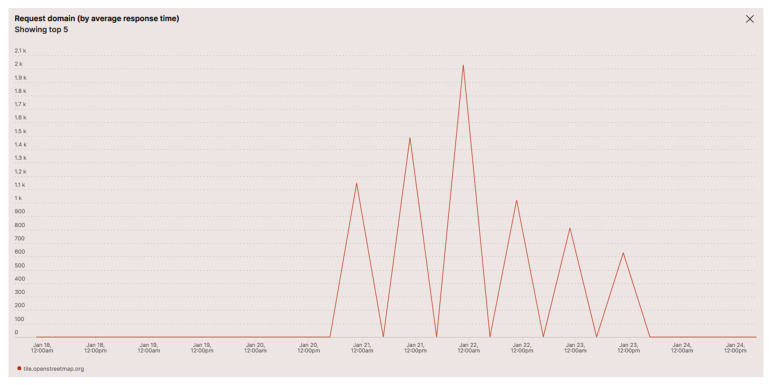
Average HTTP Response time during navigation.

**Figure A4:**
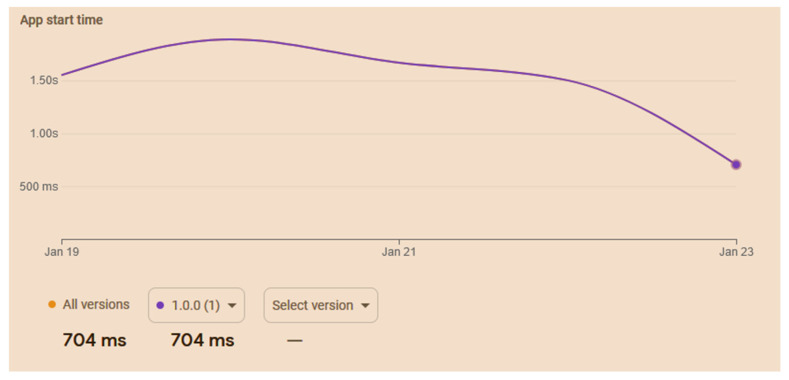
Average App Start Time.

**Figure A5:**
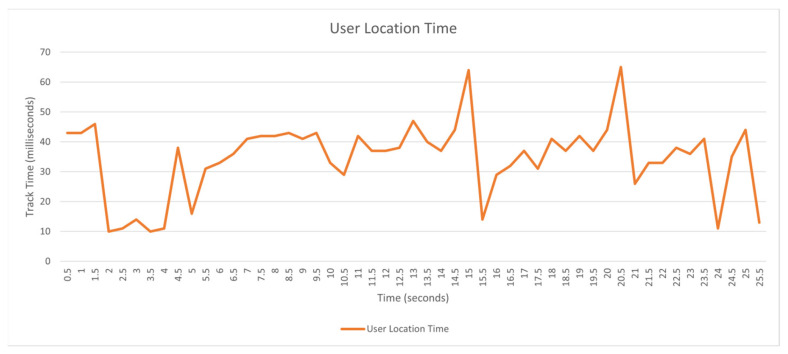
User Location Time.

## Data Availability

The data presented in this study are available on request from the corresponding author after the completion of the research project.
